# Inflammatory Cytokine Networks in Gastrointestinal Tract Graft vs. Host Disease

**DOI:** 10.3389/fimmu.2019.00163

**Published:** 2019-02-22

**Authors:** Clint Piper, William R. Drobyski

**Affiliations:** ^1^Department of Microbiology and Immunology, Medical College of Wisconsin, Milwaukee, WI, United States; ^2^Department of Medicine, Bone Marrow Transplant Program, Medical College of Wisconsin, Milwaukee, WI, United States

**Keywords:** graft vs. host disease, inflammatory cytokines, allogeneic hematopoietic stem cell transplantation, gastrointestinal tract, mouse models

## Abstract

Graft vs. host disease (GVHD) is the major non-relapse complication associated with allogeneic hematopoietic stem cell transplantation (HSCT). Damage to the gastrointestinal (GI) tract from acute GVHD is a particularly serious event that can result in significant morbidity and mortality. Proinflammatory cytokines play a critical role in the pathophysiology of intestinal GVHD, in part by activating donor T cell populations which subsequently induce tissue damage. In this review, we summarize pre-clinical data derived from experimental murine models that have examined the role of inflammatory cytokine pathways that play critical roles in the pathophysiology of GVHD of the GI tract. Specific areas of focus are on STAT 3-dependent cytokines (e.g., IL-6, IL-23, and IL-21), and members of the IL-1 cytokine family, both of which have been shown to induce pathological damage within the GI tract during this disease. We also review established and ongoing efforts to translate these pre-clinical findings into the clinic in an effort to reduce morbidity and mortality due to this complication.

## Graft vs. Host Disease

Graft-vs.-host disease (GVHD) is the major complication that occurs after allogeneic hematopoietic stem cell transplantation (HSCT) and is the leading cause of transplant-related mortality (1, 2). Mature T cells, which are present in the donor stem cell graft, are instrumental in the development of GVHD in HSCT recipients (1, 2). These pathogenic T cells are activated and clonally expand in response to recognition of a cognate recipient-derived peptide on an antigen presenting cell (APC), mounting an adaptive immune response against healthy recipient tissues. Current evidence in the literature suggests that two phases of antigen presentation occur (3). GVHD is initiated by recipient hematopoietic and non-hematopoietic APCs by a process termed direct alloantigen presentation (4, 5). Following the elimination of recipient-derived APCs post-transplantation, donor-derived APCs sustain GVHD by presenting recipient-derived peptides through the indirect pathway (6–9). Studies have revealed that the most important donor-derived APCs in this process are classical dendritic cells (7, 9).

GVHD has been divided into two phases, termed acute and chronic, which are distinguishable based on the timing of onset as well as unique clinical and pathological manifestations (10–12). During the acute phase, which is responsible for significant mortality (13), GVHD targets a restricted set of organs including the skin, gastrointestinal (GI) tract, lung, and liver (1, 2, 4). Compelling data in experimental models have shown that the GI tract plays a primary role in the propagation of this disease (14, 15). Damage to the gastrointestinal mucosa from the conditioning regimen results in the release of damage- and pathogen-associated molecular patterns (DAMPs and PAMPs) (16, 17), which activate cells of the innate immune system through the ligation of pattern recognition receptors (PRRs) (2). This ultimately leads to the generation of clonally-expanded alloreactive T cells which mediate further damage, creating an inflammatory cascade (18). From a clinical perspective, involvement of the GI tract is a major cause of morbidity and can result in significant complications including protracted diarrhea, requirement for parenteral nutrition, and infectious complications due to translocation of bacteria across a damaged mucosal barrier (19). Given the pivotal role that the GI tract plays in acute GVHD biology, strategies designed to reduce inflammation in this target organ have the potential to significantly decrease morbidity and mortality associated with this disease.

## STAT3 Signaling in GVHD of the GI Tract

During the formation of an immune response, the transduction of signals from the T cell receptor, costimulatory ligands, and cytokines into the nucleus is required for the differentiation of naïve T cells into effector lineages. During GVHD, these effector alloreactive cells are then able to secrete inflammatory cytokines and acquire cytotoxic capability, leading to tissue damage. Signal transducer and activator of transcription (STAT) proteins are responsible for much of these gene expression changes as a result of signaling through cytokine receptors. Upon ligation of a cytokine to its receptor, Janus kinases (JAKs) bind to the cytoplasmic domain of the receptor and become active, phosphorylating the appropriate STAT proteins, which then dimerize and translocate to the nucleus where they execute their function in driving transcriptional changes (20). Of these STAT proteins, STAT3 is particularly important in T cell pathogenicity during GVHD (21). In fact, treatment with a small molecule that inhibits STAT3 phosphorylation (22) or transplantation with STAT3-deficient T cells (21) significantly reduced GVHD-related mortality and pathological damage within the colon, providing support for the premise that STAT3-depednent cytokines play a prominent role in the induction of inflammation within this tissue site. Abrogation of STAT3 signaling was associated with a reduction in donor effector T cells with a corresponding increase in the number of regulatory T cells (Tregs). These results suggested that the STAT3 signaling pathway plays a critical role in balancing the effector and regulatory arms of the immune system within the context of GVHD. This basic premise has been confirmed *in vitro* using human cells where small molecule inhibition of STAT3 signaling suppressed alloreactive T cell proliferation while enhancing expansion of induced Tregs (iTregs) (23). In addition, CD4^+^ T cell STAT3 activation has been associated with an increase in T_H_17 cells and corresponding pathological damage within the GI tract in patients (23).

In contrast to the proinflammatory nature of STAT3 signaling in alloreactive T cells, expression of STAT3 in recipient myeloid cells was found to exacerbate GVHD (24). Notably, this analysis was limited to LysM-expressing cells, which are predominantly of the macrophage/monocyte lineage. While this study did not explore a mechanism for why STAT3 signaling in recipient myeloid cells elicits a paradoxical anti-inflammatory effect, the authors did note an increase in the number of donor CD4^+^ and CD8^+^ T cells in the spleen and an elevation in serum IFN-γ and IL-17 in LysM-Cre STAT3^fl/−^ recipients compared to WT recipients, suggesting that this subset of recipient myeloid cells might indirectly regulate donor T cell responses. Interestingly, deficiency in donor myeloid cells had no impact on overall GVHD severity (24). Thus, the proinflammatory effects of STAT3 signaling appear to be mediated through T cells and not myeloid cell populations. The potential clinical significance of these observations derives from the fact that a number of the inflammatory cytokines that have been implicated in the pathophysiology of GVHD, specifically within the gastrointestinal tract, use STAT3 as part of their signaling pathway, and therefore are amenable to blockade with appropriate and specific antibodies. The STAT-dependent cytokines which have been most critically examined with respect to GVHD within the GI tract are IL-6, IL-23, and IL-21.

## Interleukin 6

IL-6 is a proinflammatory cytokine that is crucial in initiating a T_H_17 immune response. In the presence of IL-6 and TGF-β, naive T cells are able to differentiate into cells of the T_H_17 lineage, whereas in the absence of this cytokine, these same cells are directed to become Tregs (25, 26). Specifically, TGF-β-induced Foxp3 is able to inhibit the transcriptional activation of RORγt which prevents the differentiation of T_H_17 cells from naïve CD4^+^ T cells (27). Thus, IL-6 appears to have a pivotal role in facilitating inflammatory responses by the immune system. In experimental murine studies, IL-6 and soluble IL-6R levels have both been shown to be increased in the gastrointestinal tract during GVHD (28). Moreover, blockade of IL-6 signaling by the administration of an antibody that binds to the IL-6 receptor significantly reduces GVHD-associated mortality and, specifically, pathologic damage within the colon (28–30). In one study (28), this was attributed to a significant increase in the absolute number of Tregs that was due to augmentation of both thymic-dependent and thymic-independent Treg production. Notably, when GVHD protection was dependent solely upon the ability to generate iTregs, blockade of IL-6 signaling resulted in a reduction in GVHD severity only within the colon (30). These results support the premise that IL-6 has an important role in mediating GVH responses within this tissue site, and that inhibition of this signaling pathway serves to recalibrate the effector and regulatory arms of the immune system in the GI tract. It should be noted that augmented Treg reconstitution has not been observed in all studies (29), although this may be due, in part, to a more abbreviated anti-IL-6R antibody administration schedule that did not provide sufficient IL-6 blockade to positively affect Treg regeneration. The requirement for more protracted anti-IL-6R antibody administration to observe robust Treg reconstitution is supported by findings in a murine sclerodermatous chronic GVHD model (31).

The potential efficacy of IL-6 blockade for the treatment and prevention of GVHD has also been examined in humans. This is due to the availability of tocilizumab which is a humanized anti-IL-6R antibody that has been FDA-approved for the treatment of patients with rheumatoid and juvenile arthritis (32, 33). Off label use of tocilizumab has therefore been possible in HSCT patients. Initial studies using tocilizumab have been in patients with steroid refractory (SR) GVHD. A total of three studies comprising 31 patients have reported results on the use of tocilizumab for the therapy of SR acute GVHD (34–36). In nearly all patients (i.e. 30/31), treatment was instituted for disease involving the lower GI tract. In two of the three studies, response rates (PR and CR) were quite similar (67 and 69%, respectively). In a third trial, however, responses were observed in only 44% of patients and were short-lived. One potential explanation for the discrepancy in these results is that the majority of patients in this latter trial had concurrent liver GVHD, and tocilizumab has not been shown in any study to have any efficacy for the treatment of disease in the liver. The reasons for this are not entirely clear, although the fact that one of the primary side effects of tocilizumab is transaminitis suggests that this agent may induce some degree of liver inflammation which could be deleterious in the setting of concurrent liver GVHD.

Inhibition of IL-6 has also been examined for the prevention of acute GVHD in allogeneic HSCT patients. The first report was by Kennedy et al. (37) who treated 48 patients (median age 48) with a single dose of tocilizumab on the day prior to transplantation in addition to standard immune suppression consisting of tacrolimus and methotrexate. The primary end point of the study was grade 2–4 acute GVHD at day 100. Conditioning was with either total body irradiation and cyclophosphamide (myeloablative) or fludarabine and melphalan (reduced intensity) and patients were transplanted with stem cell grafts from either HLA-matched sibling or matched unrelated donors. The incidence of grades II-IV and III-IV acute GVHD at day 100 was 12 and 3%, respectively, which was lower than historical controls, although this was not a randomized trial nor were the patients demographically matched to a historical or contemporaneous cohort. Of note, GVHD in the GI tract occurred in only 8% of patients and it was not specified as to whether this involved the lower or upper GI tract. Therefore, it is possible that the incidence of lower tract GI GVHD was even lower which is noteworthy given studies that have shown that upper GI tract GVHD is generally responsive to modest doses of steroids and does not impact overall survival (38, 39). Immune reconstitution was also preserved in these patients which suggested that blockade of IL-6 signaling did not deleteriously impact overall immunity (37). Flow cytometric and gene expression analysis of both monocytes and CD4^+^ T cells of patients treated with Tocilizmab revealed that there was a reduction in STAT3 phosphoyrylation and an attenuation in expression of STAT3-driven genes (37), demonstrating that IL-6 is a prominent inducer of this signaling cascade in human patients during GVHD.

A more recent study (40) also administered tocilizumab in addition to tacrolimus and methotrexate for the prevention of GVHD in an older aged population (median age 66) who underwent reduced intensity or myeloablative stem cell transplantation. All patients received busulfan-based conditioning which distinguished this trial from the previous publication. The tocilizumab administration schedule, however, was identical to that employed in the study of Kennedy et al. The cumulative incidences of grades II-IV and III-IV acute graft vs. host disease were 14 and 3% at day 100 which was similar to that observed in the prior trial. Importantly, we observed that there were no cases of GVHD of the lower gastrointestinal tract within the first 100 days. To provide additional context to these results, the authors obtained a control population from the database of the Center for International Blood and Marrow Transplant Research consisting of patients who were demographically matched for age, performance status, conditioning regimen, disease, and donor type, but who had received only tacrolimus and methotrexate for GVHD prophylaxis. This analysis revealed a lower cumulative incidence of grades II-IV acute graft vs. host disease (17 vs. 45%) and a significant increase in grades II-IV acute graft vs. host disease-free survival at 6 months (69 vs. 42%) in patients who were treated with tocilizumab, tacrolimus, and methotrexate. Collectively, these studies provided evidence that inhibition of IL-6 signaling had efficacy for the prevention of GVHD in the GI tract in allogeneic HSCT patients.

## Interleukin 23

IL-23 is a member of the IL-12 family, signals through STAT3, and is secreted by DCs, as well as other APCs such as macrophages and monocytes (41). This cytokine shares a p40 subunit with IL-12, but also contains an IL-23-specific p19 component. The p19/p40 complex binds to a heterodimer of IL-12Rβ1, which is shared with the IL-12 receptor and a unique IL-23 receptor subunit that together is present on memory/activated T cells, DCs and macrophages (42). Early studies demonstrated that IL-23 plays a critical role in disorders such as experimental allergic encephalomyelitis (EAE) (43), collagen-induced arthritis (44), and inflammatory bowel disease (45) implicating this cytokine as a pivotal mediator in the pathogenesis of inflammatory disorders and autoimmunity. Pre-clinical murine BMT studies have demonstrated that IL-23 has a selective role in the promotion of inflammation within the colon during acute GVHD. In addition, this cytokine also functions as a critical mediator linking mucosal injury and LPS translocation that occurs as a consequence of the conditioning regimen to subsequent proinflammatory cytokine production and GVHD-associated pathological damage (46, 47). In these murine models, transplantation of IL-23–deficient marrow grafts or the administration of a p19-specific antibody resulted in a significant amelioration in the severity of acute GVHD. This was shown to be due to the preferential reduction in colonic GVHD-induced pathology, accompanied by a decrease in proinflammatory cytokine production within this target organ. Donor, as opposed to host, APC production of IL-23 was demonstrated to be crucial for inducing GVHD-associated inflammation in the colon. These findings established that IL-23 has a novel organ-specific role in GVHD biology within the context of a broader systemic inflammatory disorder. Further mechanistic studies revealed that the proinflammatory effects of IL-23 were mediated at least in part by IFN-γ, not IL-17, and that an intact upstream LPS/TLR4 signaling pathway was required for IL-23-mediated colonic inflammation. Moreover, blockade of this pathway did not abrogate the graft vs. leukemia effect when tested in both acute and chronic models of leukemia (46).

More recent studies have shown that blockade of the IL-23 receptor using either genetic or antibody-based approaches similarly protect mice from lethal GVHD and pathological damage in the colon (47). In the course of these studies, a unique colitogenic CD4^+^ T cell population was identified that constitutively expresses the β2 integrin CD11c, has a biased central memory phenotype, possesses innate-like properties by gene expression analysis, and has augmented expression of the gut-homing molecules, α4β7 and CCR9. Adoptive transfer of these cells resulted in increased overall mortality, proinflammatory cytokine production, and pathology specifically in the colon. The pathogenicity of these cells was critically dependent upon co-expression of the IL-23 receptor. The fact that these CD4^+^ T cells possess an innate-like transcriptional signature suggests that they are positioned at the interface of the innate and adaptive immune systems where they are able to mediate early inflammatory events during GI tract GVHD.

There are currently two p19-specific antibodies (i.e., guselkumab and tildrakinumab) that have received FDA approval for the therapy of psoriasis (48, 49). However, specific blockade of the IL-23 signaling pathway has not been examined for the prevention or treatment of GVHD in humans. Ustekunimab which binds to the p40 subunit and thereby inhibits both IL-12 and IL-23 has been administered to patients for the prevention of GVHD in a randomized, placebo-controlled study (50). The results of this trial showed that patients treated with Ustekunimab had no difference in the incidence of grades 2–4 acute or chronic GVHD, and there was no specific protective effect on the severity of GVHD within the GI tract. Interestingly, despite the lack of effect on GVHD, there was a significant reduction in transplant-related mortality which translated to an improvement in survival. However, since the trial was not powered to assess these clinical outcomes and therefore were not the primary endpoints of the trial, the significance of these findings is not entirely clear.

## Interleukin 21

IL-21 is produced by CD4^+^ T cells, CD8^+^ T cells, and NKT cells, while the receptor for IL-21 is expressed on T cells, B cells, NK cells, dendritic cells macrophages, and epithelial cells (51). The role of IL-21 in the biology of GVHD has been examined in a number of murine transplantation models (52–55). A common finding in all of these studies has been that blockade of IL-21 signaling by either antibody-based strategies or genetic approaches is able to significantly reduce the severity of GVHD. In some instances, this was shown to be due to an increase in the reconstitution of Tregs accompanied by a commensurate reduction in the expansion of donor effector T cells, suggesting that blockade of IL-21 signaling recalibrates the effector and regulatory arms of the immune system, similar to IL-6 blockade. Notably, several studies have confirmed that blockade of IL-21 signaling results in decreased pathological damage specifically within the GI tract (52–55). More recently, tissue from the GI tract of patients with active GVHD also revealed increased IL-21 expression in mononuclear cells in the colon when comparted to samples obtained from patients with no GVHD (56), suggesting that IL-21 may play a role in gastrointestinal GVHD in humans as well. As of yet, however, there have been no trials examining whether blockade of IL-21 signaling is able to reduce the severity of GVHD in humans.

## Interleukin 1 Family Members

Interleukin-1 (IL-1) was one of the first cytokines described and was named for the soluble product of macrophages during inflammation (57). Since then, IL-1 has been discovered to be not a single gene product but a family of cytokines (58), collectively referred to as the “IL-1 superfamily” (59, 60). Most of the genes in the IL-1 family are clustered together on the same chromosome, likely attributable to a gene duplication event that is evident in their similarity in sequence, structure, and function (61). IL-1 family cytokines direct a host of events in the immune system ranging from acute inflammatory processes initiated by the cells of the innate immune system (57), to T cell differentiation (62), to the regulation of inflammation (63, 64). In this section, we will focus on the “classical” IL-1 cytokines (IL-1α and IL-1β) as well as the IL-1 receptor antagonist (IL-1RA), which binds the IL-1 receptor and blocks binding of other ligands but elicits no signaling itself (64). Furthermore, we will discuss new insights into the role of IL-33, another IL-1 family member, and its cognate receptor ST2 in mediating inflammation in the GI tract during GVHD.

## Interleukin 1

A role for the IL-1 family in the biology of GVHD was first postulated in 1991 when increased levels of IL-1α mRNA was detected in the skin of GVHD recipients in an MHC-matched, minor antigen mismatched murine transplant model (65). In addition, the administration of a recombinant human IL-1Ra was found to significantly increase survival in transplant recipients (66), supporting a role for this cytokine in the pathophysiology of this disease. Subsequent studies yielded more conflicting results, raising the issue as to whether this observed variability might, to some extent, be model-specific (66, 67). These preclinical studies, however, did lay the foundation for human trials testing the efficacy of IL-1 blockade which were conducted in patients with steroid-refractory GVHD. In a phase I/II trial, which involved a 7 day continuous infusion of recombinant IL-1Ra, 10 out of 16 patients had an overall reduction in GVHD grade, including 8 out of 11 patients with GI-tract involvement who showed improvement (68). In another phase I/II trial using a recombinant IL-1 decoy receptor, eight of 14 patients had an overall reduction in GVHD grade, with 2 of 6 patients with GI tract involvement showing organ-specific improvement (69). The largest clinical trial examining anti-IL-1-directed therapy was conducted nearly a decade after the initial observation in murine models (70). This was a double-blind, placebo-controlled trial involving 186 patients in which the study arm consisted of treatment with an IL-1 receptor antagonist for GVHD prophylaxis. The primary endpoints of this study were event-free survival, overall survival, and incidence of GVHD. Unfortunately, patients that received IL-1Ra treatment had no improvement in any of these outcome measures. Notably, recombinant IL-1Ra was administered in the peritransplant period, with IL-1Ra levels in the serum returning to baseline by day 14 (70), before most patients develop acute GVHD. Whether this administration schedule may have been responsible for the lack of any perceived effect is not clear and the ability of IL-1 blockade to prevent GVHD within the GI tract in humans remains unproven.

## Inflammasome Signaling

With the discovery of the inflammasome (71) there has been renewed interest in the role of IL-1 cytokines in gastrointestinal GVHD. This is due to the fact that the GI tract is a source of innate immune activating pathogen- and damage-associated molecular patterns (15), some of which can cause activation and assembly of the inflammasome. In the case of one particular NLRP3, the ligation of TLR4 by lipopolysaccharide (LPS) causes the up regulation of inflammasome substrates and components such as pro-IL-1β and NLRP3 (72), as well as the deubiquitination and stabilization of NLRP3 (73). As a complementary step, the ligation of the purinergic receptor P2X7 by ATP provides the second step in inflammasome activation, ultimately leading to the assembly of NLRP3 with ASC, cleavage of pro-caspase-1, and caspase-1-mediated conversion of pro-IL-1β to its secreted and biologically active form (73).

Jankovic and colleagues provided the first evidence that the NLRP3 inflammasome is important in the pathophysiology of gastrointestinal GVHD (74). This report showed that in a MHC-mismatched murine model, pretransplant but not post-transplant treatment of mice with an IL-1 receptor antagonist or an IL-1β blocking antibody prevented GVHD. Furthermore, they showed that the NLRP3 inflammasome was required for production of IL-1 in the GI tract and corresponding lethality post-transplant. Biopsies from patients with GVHD had a higher proportion of cleaved (active) caspase-1 staining cells by immunohistochemistry compared to biopsies from transplant patients without GVHD. PBMCs isolated from patients with GVHD produced more IL-1β than those from BMT patients without GVHD and healthy controls. These results provide strong evidence that the NLRP3 inflammasome/IL-1β pathway is involved in the pathogenesis of gastrointestinal GVHD and that further investigation into these pathophysiological mechanisms is warranted to determine whether this will have potential therapeutic implications. In this regard, it is noteworthy that transplantation of microRNA(mir)-155-deficient dendritic cells has also been shown to cause less GVHD in the GI tract due to defective inflammasome activation (75). This observation could potentially be exploited clinically using antagomir administration to inhibit mir-155.

## Interleukin 33/ST2 Axis

One of the more recently identified pathways in GVHD biology is the IL-33/ST2 axis. IL-33 is a member of the IL-1 superfamily that has been identified as an alarmin and is released during cell injury and necrosis to initiate the immune response (76). IL-33 is produced primarily by a variety of non-hematopoietic cells which include endothelial cells, fibroblasts, and epithelial cells in the intestines and bronchi. Within the GI tract, in particular, IL-33 is expressed by α-SMA^+^ subepithelial myelofibroblasts which have also been termed pericryptal fibroblasts (77). Release of IL-33 leads to binding to its membrane receptor, ST2, which is expressed on a large number of immune cells (i.e., T_H_2 cells, regulatory T cells, type 2 innate lymphoid cells, macrophages, and granulocyte populations) (78). Notably, a soluble form of the receptor, sST2, lacks transmembrane and cytoplasmic domains and serves as a decoy receptor to IL-33, hampering the ability of this cytokine to elicit and effect in target cells (76).

In preclinical studies, IL-33 and sST2 have both been shown to be increased in the blood of mice during GVHD (79, 80). IL-33 is specifically increased in the GI tract where it is produced by non-hematopoietic cells in both murine models and patients with stage IV acute GVHD (79). The mechanism by which the IL-33/ST2 axis manipulates GVHD is complex. Transplant recipients that are deficient for IL-33 are protected from GVHD and administration of IL-33 in the early post transplantation period (days 3–7 post-transplant) was shown to exacerbate this disease (79), suggesting a proinflammatory role. Post-transplant IL-33 administration was associated with reduced survival, higher serum TNF-α, and an increased number of infiltrating intestinal donor T cells (79). Of note, post-transplantation blockade of IL-33 with an sST2-Fc receptor fusion protein also attenuated GVHD. In contrast, peri-transplant IL-33 administration (days −10 to +4) attenuated disease (81). This reduction in GVHD severity was associated with an increase in recipient mST2^+^ regulatory T cells, a cell population that is able to survive after total body irradiation. A reduction in GVHD was also observed in mice that received peritranspant sST2 blockade, which potentiates the effects of IL-33 due to an inhibition of its decoy receptor (80). Notably, sST2 blockade did not affect expression of mST2 on specific lymphocyte populations, such as T_H_2 cells and regulatory T cells (80). These data indicate a paradoxical role for IL-33/ST2 signaling in GVHD, whereby early treatment of IL-33 downregulates inflammation in the GI tract, whereas administration of IL-33 is proinflammatory once disease has been established. Thus, the pro or anti-inflammatory effects of IL-33 in GVHD appear to be highly schedule dependent. While the reasons for this are still not completely clear, this disparity could be due to differences in cell populations which populate the gastrointestinal tract at various stages post-transplantation. For example, recipient Tregs of which there is an ST2^+^ subset which can be expanded with peritransplant IL-33 treatment are present early post-transplantation and can suppress GVHD (81). In fact, the depletion of Tregs during peritransplant IL-33 administration results in the loss of protection against GVHD (81). Conversely, IL-33 signaling appears to act primarily on ST2^+^ conventional T cells later post-transplantation resulting in an exacerbation of the disease (79). The temporal effects of IL-33 in these models may also be, to some extent, dose-dependent, as mice which received IL-33 peritransplant and were protected from GVHD received more than twice the dose over a much longer treatment window (81) than those that received posttransplant IL-33 (79).

In allogeneic HSCT patients, ST2 has emerged as a powerful biomarker that is predictive for GVHD severity in patients. Specifically, biomarker panels obtained early post transplantation which incorporated ST2 have been shown in several studies to be predictive for increased non-relapse-related mortality (82–84). In addition, in one of these studies (83), severe GI tract GVHD was also significantly greater in those patients who were in the cohort with highest levels of ST2. ST2 has also emerged as a critical component of a biomarker panel that has been shown to be predictive for response or lack thereof in steroid refractory acute GVHD (85). This disease carries a particularly high mortality which is typically attributable to disease involving the GI tract which is often the proximate cause of death. To date, there have been no clinical studies which have attempted to interrupt signaling through the IL-33/ST2 pathway in order to reduce inflammation. However, recent efforts to develop small molecule inhibitors that interfere with this pathway has shown promise in murine studies where there has been a reduction in sST2 plasma levels, reduced GVHD, and improved survival (86). Thus, these data provide hope that targeting of this pathway may soon be clinically feasible.

## Other Cytokines

### Granulocyte-Macrophage Colony Stimulating Factor (GM-CSF)

GM-CSF was originally characterized and designated as a hematopoietic growth factor which could promote myelopoiesis in the bone marrow. However, more recent studies have demonstrated that GM-CSF is largely redundant in the development of the hematopoietic system as mice deficient in either the cytokine or its receptor have only limited defects in steady-state myelopoiesis (87, 88). Rather, GM-CSF has been implicated as a key signaling molecule which is able to activate the innate immune system in autoimmune and proinflammatory syndromes such as experimental autoimmune encephalomyelitis (EAE) and rheumatoid arthritis (89). In EAE, for example, GM-CSF is required for the induction of autoimmunity (90, 91) and serves as a conduit between CD4^+^ T cells and CCR2^+^ macrophages (92). In the latter cell population, GM-CSF institutes a proinflammatory transcriptional program which facilitates pathological damage within the central nervous system (92). Recently, a role for GM-CSF in GVHD has been posited by Ulrich et al. who described a population of BATF-dependent IL-7R^hi^ T cells that produce GM-CSF (93). In this report, which primarily focused on inflammation in the gastrointestinal tract, GM-CSF^−/−^ T cells induced less GVHD in the colon as evidenced by reduced colonoscopy and clinical scores, as well as increased overall survival. This finding was replicated in a MHC-mismatched and haploidentical transplant models by Tughes et al. (94). Interestingly, GVHD was attenuated when GM-CSF receptor deficient bone marrow donors were used, suggesting that the proinflammatory effects of GM-CSF are at least in part mediated through donor myeloid cells. However, a mechanism or specific target myeloid populations for GM-CSF signaling were not definitively established. Thus, further studies are needed to elucidate the full role of this proinflammatory cytokine. Given that mavrilimumab (95), an anti-GM-CSF receptor alpha monoclonal antibody, and MOR103 (96), a humanized anti-GM-CSF antibody, are currently in clinical trials for the treatment of rheumatoid arthritis and multiple sclerosis, respectively, GM-CSF could represent a new target for the prevention of GVHD in the GI tract.

### Interferon-Gamma (IFN-γ)

Donor T cells with a T_H_1 cytokine phenotype have an important role in the pathophysiology of GVHD (1, 2). The signature cytokine of these cells, IFN-γ, has also been demonstrated to induce pathological damage in the GI tract during GVHD. However, inhibition of IFN-γ signaling has divergent effects on GVHD target organs in pre-clinical murine models. Specifically, these studies have shown that mice transplanted with IFN-γ deficient grafts have reduced pathology in the GI tract (14, 97), but rapidly develop an idiopathic pneumonia syndrome (IPS)-like disease early post-transplantation resulting in increased mortality (97–99). The protective effect of IFN-γ is mediated through host non-hematopoietic cells, likely the lung parenchyma itself (97, 99), which inhibit the production of IL-6 by signaling through the IFN-γR (99). In contrast, IFN-γR signaling in T cells appears to be proinflammatory. A reduction in GVHD severity was observed when recipients were transplanted with IFN-γR-deficient T cells (97, 100). Additionally, reduced pathological damage occurred in the GI tract of these recipients without a commensurate increase in tissue damage in the lung (100). This was attributable to reduced expression of the chemokine receptor, CXCR3, which altered trafficking into this tissue site. To date, likely due to the divergent effects observed with inhibition of IFN-γ signaling in these pre-clinical models, there have been no clinical studies targeting this pathway to ameliorate GVHD in the GI tract.

## Conclusions

The GI tract is the target organ which induces the most profound morbidity in patients who develop GVHD after allogeneic HSCT, and is responsible for much of the mortality associated with this disease. Inflammatory cytokines have been shown to play a pivotal role in this process and serve to amplify the pathogenic effects of alloreactive donor T cells. Ongoing research, primarily in murine models, has identified a number cytokines (IL-6, IL-21, IL-23, IL-1, IL-33, GM-CSF) and cytokine pathways (e.g., STAT3 signaling dependent, inflammasome-mediated) that are operative in the pathophysiology of GVHD of the GI tract. Notably, many of these cytokines or specific pathways can be targeted with existing, clinically available antibodies or small molecules designed to inhibit their activity in human transplant recipients. Thus, there is now optimism that the further evolution of this work will lead to the rational development of new strategies designed to reduce the severity of this complication in man and ultimately result in improved overall survival.

## Author Contributions

CP and WD wrote and edited the manuscript.

### Conflict of Interest Statement

The authors declare that the research was conducted in the absence of any commercial or financial relationships that could be construed as a potential conflict of interest.
